# Differences of Disabling Symptoms between Previously Hospitalized or Non-Hospitalized Currently Working Long-COVID Survivors One Year after Infection: A Descriptive Study

**DOI:** 10.3390/healthcare11162306

**Published:** 2023-08-16

**Authors:** Laura López-López, Andrés Calvache-Mateo, Araceli Ortiz-Rubio, María Granados-Santiago, Alejandro Heredia-Ciuró, Javier Martín-Núñez, Marie Carmen Valenza

**Affiliations:** 1 Physiotherapy Department, Faculty of Health Sciences, University of Granada, 18016 Granada, Spain; lauralopez@ugr.es (L.L.-L.); andrescalvache@ugr.es (A.C.-M.); aortiz@ugr.es (A.O.-R.); ahc@ugr.es (A.H.-C.); javimn@ugr.es (J.M.-N.); cvalenza@ugr.es (M.C.V.); 2 Nursing Department, Faculty of Health Sciences, University of Granada, 18016 Granada, Spain

**Keywords:** COVID-19, Long-COVID syndrome, SARS-CoV-2, disabling symptoms, persistent symptoms

## Abstract

This study aimed to describe the presence of disabling symptoms in currently working Long-COVID survivors by comparing the hospitalized and non-hospitalized one year after infection. Patients with Long-COVID syndrome (LCS) that have been infected by COVID-19 a year ago and were actually working were included. Participants that had been hospitalized due to COVID-19 were included in the LCS hospitalized group, and participants that had not been hospitalized were included in the LCS non-hospitalized group. The eligible patients were prompted to complete the latest self-report version of the COVID-19 Yorkshire Rehabilitation Screening Tool (C19-YRS). A total of 465 subjects were included in the study. Participants in the LCS hospitalized group were significantly older, had a significantly higher BMI, and had a significantly higher prevalence of women compared to the LCS non-hospitalized group. Additionally, participants in the LCS hospitalized group had obtained significantly worse results in symptom severity, functional disability, and global health perceived subscales of C19-YRS compared to the participants included in the LCS non-hospitalized group. We concluded that disabling symptoms are presented in patients with LCS at working age one year after infection and are higher in LCS hospitalized patients compared to LCS non-hospitalized patients.

## 1. Introduction

In 2020, the world experienced the most catastrophic event of the last century due to the pandemic of the coronavirus disease 2019 (COVID-19) caused by SARS-CoV-2 [1].

The COVID-19 pandemic has affected the health and lives of people around the world, with the potential for further effects in the future [2].

In the beginning, COVID-19 was considered as a pulmonary illness with extrapulmonary manifestations. As the pandemic spread, there has been growing evidence that COVID-19 involves multiple organs/systems. The COVID-19 clinical symptoms include fever, dyspnea, chest tightness, cough with or without sputum production, myalgia, fatigue, and nausea, among others. These symptoms have led to a surge of hospitalizations, many of which have required prolonged intensive care unit stays and mechanical ventilation [3,4].

Based on previous knowledge from the Severe Acute Respiratory Syndrome epidemic (SARS), a need for long-term health care could be predicted for COVID-19 patients after hospitalization [5]. The pathophysiological mechanisms of COVID-19, the effects of hospitalization, and the presence of sustained stress could contribute to developing persistent symptoms in survivors [6] that can last for at least 6 months after hospital discharge [7].

Additionally, hospitalization per se is defined as a transient period of recovery from an acute illness as well as an experience of generalized risk for a wide range of adverse health events. Thus, this condition may be better characterized as a post-hospital syndrome; an acquired condition of vulnerability. The theory of illness suggests that the risks after discharge might derive as much, or more, from the allostatic stress that patients experience in the hospital as they do from the lingering effects of the acute illness that precipitated the hospitalization. At the time of discharge, the physiological systems are impaired, physiological reserves are depleted, and the body cannot effectively avoid or mitigate health threats [8]. In this line, hospitalization can imply psycho-physiological effects at long term that have been reported in different populations [9,10,11,12].

Although non-hospitalized patients represent a larger patient group, most of the published data are related to hospitalized patients. Concretely, the study of Bergquist et al. (2020) [9] suggested demographic and clinical characteristics of non-hospitalized patients that differ significantly, associated with a broad range of mild-to-severe symptoms. Due to the small amount of evidence, characterizing and comparing symptoms among hospitalized and non-hospitalized patients may guide more effective approach strategies [10].

There is evidence of a second pandemic generated by all those patients that, after the acute phase of SARS-CoV-2 infection, continue to suffer long-lasting symptoms. This condition is called Long-COVID syndrome [13] or post-COVID-19 condition [14] and is generating an important burden on international health systems. The actual evidence suggests that the number of individuals suffering from COVID-19 sequelae will dramatically rise [15,16]. These patients suffer from several symptoms that continue for more than 12 weeks after COVID-19 infection and cannot be explained by an alternative diagnosis. General Long-COVID symptoms include myalgia, breathlessness, fatigue, cognitive blunting, or a combination of symptoms [17].

Long-COVID syndrome (LCS) is used to describe the persistence or appearance of one or more symptoms irrespective of the viral status. The majority of people with LCS are PCR negative and show biochemical and radiological recovery [18]. A meta-analysis published by López-León et al. [19] found that 80% of COVID-19 survivors exhibited at least one long-COVID-19 symptom, being fatigue (58%), headache (44%), attention disorders (27%), hair loss (25%), and breathlessness (24%) being the most frequent. Additionally, Fernandez-de-las-Peñas et al. [20] carried out a systematic review and meta-analysis and concluded that 45.9% of the LCS patients exhibited more than one long-COVID-19 symptom from 90 days after onset/hospitalization, with fatigue and dyspnea being the most prevalent. While there is a global recognition of the relevance of exploring therapeutic options for LCS patients, there is not enough evidence of the evolution of LCS in the long term to develop adequate therapeutic options.

Workplace has been one of the settings where SARS-CoV-2 transmission is more frequent [21], affecting global work-related patients’ profile when considering age, gender, and comorbidity characteristics [22,23,24].

Concretely, the prevalence of long-COVID symptoms is higher in younger people than older, concretely, in people among 35–49-year-olds and 50–69-year-olds, compared to the general population with COVID-19 diagnosed [25,26].

In addition to the health problems associated with their condition, actively working people with Long-COVID syndrome used to report a decrease in their quality of life and working capacity due to adverse effects and stressful situations. Fear of job loss and future job insecurity, unsafe work environments, quarantine, infection and/or spreading the infection, and COVID-19-related discrimination and/or stigma are considered additional factors that may worsen the psychological state, being a societal burden in this population [27,28].

Therefore, the effects of the COVID-19 pandemic on people of working age should be investigated in order to support safe re-entry and ensure good working conditions for all workers [29]. To date, many studies have analyzed the short-term consequences of the pandemic on health workers, both physically and psychologically. The study of Lai et al. (2019) [30] found that a significant number of healthcare workers had scores indicating symptoms of distress (71.5%) and anxiety (44.6%), where 50% had symptoms of depression, and 34% reported poor sleep. Additionally, the study of Chew et al. (2020) [31] found that the most reported physical symptoms in healthcare workers were headache (31.9%) and throat pain (33.6%).

However, no studies have been conducted to analyze the disabling symptoms of SARS-CoV-2 infection in the general working-related population [32,33]

Given the significant importance of understanding the long-term effects of COVID-19 to enhance patient care, there are numerous related articles on this subject. However, the majority of these studies focused on patients with prior hospitalization and had relatively short to medium-term follow-up [19,34]. Additionally, they have included smaller sample sizes, did not differentiate between hospitalization and non-hospitalization, and they did not include the currently working population [35,36]

Additional research is required to delve into symptom clusters, assess symptom severity, and understand how these symptoms impact daily activities. By categorizing patients based on the severity of their condition, suitable interventions and treatment strategies can be identified, and the trajectory of the disease can be mapped [37].

This study aimed to describe the presence of disabling symptoms in hospitalized and non-hospitalized workers with Long-COVID syndrome one year after infection.

## 2. Materials and Methods

### 2.1. Design

This is a descriptive cohort study carried out from November 2021 to April 2022. This study was conducted with the recommended guidelines for the design of observational studies, and the criteria and checklist of Strengthening the Reporting of Observational Studies in Epidemiology (STROBE) were applied [38]. We conducted this study in accordance with the Declaration of Helsinki 1975, revised in 2013 [39]. Ethical approval for this study was obtained from the Biomedical Research Ethics Committee of Granada.

### 2.2. Participants

Participants were recruited from the Andalusia patient Long-COVID association. The inclusion criteria were the following: (1) people actively working that were diagnosed with COVID-19 a year ago, (2) with Long-COVID syndrome that meets the World Health Organization’s definition for this disease [5], (3) whose age is over 18 years, and (4) who signed the informed consent. Patients were excluded if they had cognitive impairments that prevented them from understanding and answering, if they did not want to participate, if they suffered reinfection with SARS-CoV-2, or if they did not sign the informed consent. All participants were evaluated after the first dose of the COVID-19 vaccination, with Omicron and Delta being the main variants of SARS-CoV-2 in Spain [40,41].

### 2.3. Group Assignment

Once the inclusion and exclusion criteria were verified and the informed consents were signed, participants were divided into two groups based on the presence of hospital admission-related COVID infection. Participants who had been admitted to the hospital due to COVID infection were included in the Long-COVID syndrome hospitalized group, and participants that had not been admitted to the hospital due to COVID infection were included in the Long-COVID syndrome non-hospitalized group.

### 2.4. Survey Questionnaire

The eligible patients were prompted to complete a cross-sectional survey that includes anthropometric and sociodemographic data that included age, sex, the body mass index, and time since infection. For patients belonging to the Long-COVID syndrome hospitalized group, the length of the hospital stay and the length of the Intensive Care Unit were also recorded.

Additionally, participants in both groups had to complete the latest self-report version of the COVID-19 Yorkshire Rehabilitation Screening Tool (C19-YRS).

The C19-YRS was developed by a rehabilitation team as a measure of the impact of SARS-CoV-2 infection. It provides insight into patients’ needs and allows for periodic reassessments to monitor patients. The scale has been widely used to measure symptomatic severity and disabling symptoms and is recommended by the UK’s National Health Service England [42] and the National Institute for Health and Care Excellence (NICE) [43].

The C19-YRS was the first validated scale reported in the literature for patient assessment and monitoring in Long-COVID syndrome. It consists of a 22-item scale that contains four subscales measuring symptom severity, functional disability, overall health, and additional symptoms. Participants were asked to rate their functionality (both before and after having COVID-19) including breathlessness, throat and airways, ability to engage in everyday activities, fatigue, cognitive functioning, distress, anxiety, and depression. Multiple items are responded to with yes/no and some symptoms or functional abilities are rated by the respondent on a scale from 0 (having no presence of symptom) to 10 (being most severe and life disturbing). The overall health status is also captured on a 0–10 numerical rating scale (NRS) [44]. “How good or bad is your health overall now and pre-COVID?” and “Have you developed any changes in the sensitivity of your throat such as troublesome cough or noisy breathing?” are questions included in this questionnaire. The C19-YRS outcome measure of Long-COVID patients has satisfactory psychometric properties and internal consistency was high (Cronbach’s α = 0.891) [45]. The C19-YRS is free of charge and can be obtained under license from the University of Leeds (https://licensing.leeds.ac.uk/product/c19-yrs-covid-19-yorkshire-rehabilitation-scale, accessed on 12 April 2022).

The C19-YRS was self-administered by the patients themselves at home one year after the COVID-19 infection. Once the C19-YRS assessment was completed, the researchers transferred each patient’s data into an Excel spreadsheet that was completely anonymized.

### 2.5. Statistical Analysis

Statistical Package SPSS version 23.0 (International Business Machines, Armonk, NY, USA) was used to analyze the data obtained. The nominal data were expressed as frequency (percentage). Continuous variables were presented as mean and standard deviation (SD). Before statistical analysis, the Kolmogorov–Smirnov test was performed to assess the normality of the variables. Descriptive statistics were carried out to describe sample baseline characteristics. The within-group and between-group comparison was performed after subjects were grouped by hospitalization. For nominal variables, the Chi-square test was used to identify the differences between groups. Normally distributed continuous variables were compared with the Student’s *t*-test. A 95% confidence interval was used for statistical analysis. Statistical significance was accepted at a *p*-value of 0.05.

## 3. Results

**Figure 1:**
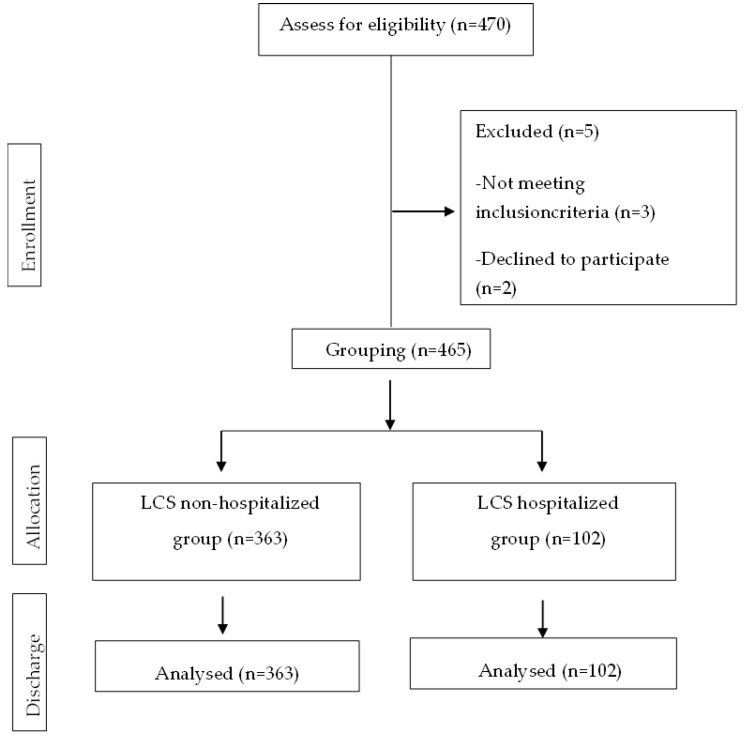
CONSORT Flow Diagram.

Finally, from the 470 potential patients, a final sample size of 465 was selected in the study; 102 participants were included in the Long-COVID syndrome hospitalized group, and 363 participants were included in the Long-COVID syndrome non-hospitalized group. (Figure 1).

The characteristics of the sample are presented in Table 1.

As we can see in Table 1, there were significant differences in the mean age, BMI, and sex prevalence between groups. Participants in the LCS hospitalized group were older (mean age 47.35 ± 8.53) and overweight (26.66 ± 6.31) compared to the participants in the LCS non-hospitalized group, where they were younger (mean age 43.65 ± 9.57) and were of normal weight (24.73 ± 5.74). Additionally, the LCS hospitalized group had a higher percentage of female patients, being statistically significant. No significant differences in time since infection were found between the two groups.

Table 2 shows symptoms severity, functional disability, and global health perceived between groups.

As seen in Table 2, both groups were similar in all the C19-YRS subscales before infection (*p* > 0.05). In the within-group comparison, the results show statistically significant differences (*p* < 0.001) in all subscales one year after infection. Participants in the LCS hospitalized group have obtained significantly worse results in symptoms severity, functional disability, and global health perceived compared to the participants included in the LCS non-hospitalized group. The aftermath that has brought the highest punctuation has been fatigue, breathlessness, and pain with the LCS hospitalized group showing worse results. In addition, patients hospitalized during the acute phase show greater impairment of usual activities and lower scores for perceived global health.

In Table 3, additional symptoms are presented in both groups.

It can be observed that participants in the LCS hospitalized group have shown a higher prevalence of additional symptoms compared to the participants included in the LCS non-hospitalized group, which is significant for all of them except for nutritional alterations and bowel and bladder incontinence (*p* > 0.05). Moreover, the most prevalent in both groups were concentration, memory, communication, and labor difficulties.

## 4. Discussion

This study aimed to better define the presence of disabling symptoms in hospitalized and non-hospitalized patients with Long-COVID syndrome at working age one year after infection. With regard to the characteristics of the sample, the results of this study show that patients hospitalized during the acute phase of the disease were older, had a higher body mass index, and were more often female. With respect to disabling symptomatology, the results of this study have shown that patients with Long-COVID syndrome of working age have disabling symptoms one year after SARS-CoV-2 infection, with this symptomatology being worse in patients who have been hospitalized during the acute phase of the disease. Specifically, the symptomatology that showed the greatest severity among patients was fatigue, breathlessness, and pain, with these results being worse in the group of previously hospitalized patients. In addition, previously hospitalized patients showed greater impairment of usual activities and perceived global health.

Although several studies [46,47] reported a greater presence of disabling symptoms among hospitalized patients during the acute phase of SARS-CoV-2 infection, no studies have been carried out in hospitalized and non-hospitalized working-age patients one year after infection. In our study, we found that fatigue, breathlessness, and pain were the most disabling symptoms in both groups one year after COVID-19 infection. Regarding the fatigue presented by these patients, it is clearly related to the underlying pulmonary pathology, but in addition, there is a central phenomenon present in post-viral fatigue, which is mediated by inflammation and neurocognitive dysfunction [48]. The mechanisms that generate dyspnea involve not only changes in the lung parenchyma, but also include deconditioning, cardiovascular dysfunction, and dysfunctional breathing [49]. Pain in these patients is due to the proinflammatory response over time caused by the immune system’s response to SARS-CoV-2 infection and the negative biopsychosocial factors because of the pandemic situation [50].

In addition, hospitalized Long-COVID patients showed a worse general state, highlighting the incidence of cognitive problems (concentration, memory, communication, and labor difficulties). Several studies [51,52] have objectively evaluated the cognitive deficits in Long-COVID patients at 12 months post-infection. Douaud et al. [51] observed a greater reduction in grey matter thickness, tissue damage in regions that are functionally connected to the primary olfactory cortex, and a greater reduction in global brain size in the non-hospitalized SARS-CoV-2 patients. Besides, Diez-Cirarda et al. [52] showed structural and functional brain abnormalities 11 months after the acute infection of COVID-19. These abnormalities could be associated with cognitive dysfunction in post-COVID syndrome.

Pain has been previously studied in post-COVID patients. Khoja et al. (2022) [53] concluded that musculoskeletal pain was one of the most common symptoms in post-COVID patients. In a descriptive study of pain in survivors of COVID-19, the authors found a 19.6% of de novo chronic pain prevalence that interfered with their ability to function. Their pain was located primarily in the head and neck, but frequently occurred in the lower limbs and often moved around the body [54]. In the same line, Calvache-Mateo et al. (2023) [55] carried out an observational study with the objective of evaluating the characteristics of pain (i.e., pain intensity, pain interference, and clinical presentation) in Long-COVID-19 patients, comparing the location of pain between those of successfully recovered COVID-19 and healthy matched controls. They found that Long-COVID syndrome patients obtained significantly higher pain intensity and interference. Additionally, they concluded that the pain of these patients is characterized by widespread location with the most frequent locations being the neck, legs, and head, and which significantly affects the quality of life of these patients.

Fernández-de-Las-Peñas et al. [13] carried out a systematic review and meta-analysis and concluded that fatigue and dyspnea were the most prevalent long-COVID-19 symptoms in hospitalized and non-hospitalized patients, particularly at 60 and ≥90 days of follow-up. In addition, they highlighted the importance in carrying out studies with longer time frames because it should be considered that symptoms appearing before 12 weeks after recovery from acute infection have been considered as post-acute sequelae of COVID-19 and not as the real long-COVID-19 syndrome [1]. The descriptive studies carried out to date regarding pain show a moderate intensity of pain, similar to that presented by the patients in this study; however, these studies did not have a follow-up period of one year [54].

Morin et al. [56] carried out a study to determine the consequences at 4 months in patients hospitalized for COVID-19. They found that, of the 471 patients included, 51% reported at least one symptom that did not exist before COVID-19 infection, with fatigue (31.1%) and memory difficulties (17.5%) being the most prevalent. In our study, we have found similar results one year after infection. Additionally, after differentiating according to the hospitalization, we have observed that fatigue, concentration, and memory difficulties are more disabling in the hospitalized group compared to the non-hospitalized group. In previous studies, results similar to ours were observed in terms of concentration and memory problems in young patients, which can interfere with their work abilities [57,58].

The immunological processes behind the persistence of COVID-19 symptoms have been broadly investigated and discussed [59]. Several lines of evidence indicate that neuropsychiatric disturbances in Long-COVID patients may be associated with a hyperinflammatory state with elevated levels of pro-inflammatory cytokines, such as IL-6, IL-2, IL-17, the granulocyte-colony stimulating factor, and TNF-α [60]. Moreover, a high level of antinuclear antibodies found in Long-COVID subjects suggests autoimmune etiology of neurocognitive symptoms [61].

Our study observed a worse global health status in hospitalized Long-COVID patients in accordance with previous studies [62,63,64]. The majority of Long-COVID syndrome participants in this study were females that had not been hospitalized. It should be considered in the management of this population. The majority of the studies about Long-COVID included hospitalized patients, but the reality is that most of these patients have not been hospitalized. It is important to take this into account, not only to carry out more research about LCS non-hospitalized patients, but also that these patients should be properly treated in health systems.

The sum of disabling physical symptoms, together with the cognitive symptoms of working-age patients with SCI, can considerably impair their work performance, which can be the cause of certain negative psychological factors such as anxiety, depression, and stress, as well as certain negative social factors due to the fear of losing their job, financial difficulties, fear of reinfection, etc. [26].

Thus, it is necessary to develop multidisciplinary treatment programs that understand the multifactorial etiology of this pathology and address the specific needs of patients. In addition, there is a need for the development of employment policies that allow patients with SCI of working age to return to their usual work environment in a safe environment adapted to their current needs as they recover, limiting the risk of re-infection.

Some limitations of the study have to be reported. One important constraint to consider is that patients themselves reported their symptoms and their severity, introducing the possibility of some subjectivity in their documentation. In future studies, it could be complemented with other variables to provide more objectivity to the results. Another limitation of this study could be the fact that the sample was obtained from an association of patients with the post-COVID-19 syndrome, which makes the patients included more proactive in seeking help, perhaps because they have a greater intensity of symptoms. Additionally, the impact of SARS-CoV-2 infection was obtained via a questionnaire self-report. Finally, the symptoms in the acute phase of illness and the different variants of SARS-CoV-2 are not collected. For further studies, we suggest measuring this variable followed by a first face-to-face assessment.

## 5. Conclusions

Identification of the COVID-19 aftermaths will be crucial for healthcare professionals. This study has revealed that the prevalence of disabling symptoms is higher in Long-COVID syndrome hospitalized patients compared to Long-COVID syndrome non-hospitalized patients at working age a year before infection. The most disabling symptoms were fatigue, breathlessness, and pain in both groups being severe in Long-COVID syndrome hospitalized participants. Additionally, health-related quality of life and usual activities, as well as concentration, memory, communication, and labor difficulties, were also prevalent in both groups. These findings have important implications for the development of future interventions to improve the management of these patients.

## Figures and Tables

**Table 1:** Characteristics of the sample.

Variables	LCS Non-Hospitalized Group (n = 363)	LCS Hospitalized Group (n = 102)	*p*
Mean age (years)	43.65 ± 9.57	47.35 ± 8.53	<0.001 **
BMI (kg/m^2^)	24.73 ± 5.74	26.66 ± 6.31	0.003 *
Sex n (% women)	316 (87.05)	79 (77.45)	0.017 *
Time since infection (months)	12.25 ± 4.21	12.10 ± 4.36	0.753
Length of hospital stay (days)	-	14.11 ± 15.08	-
ICU n (Yes%)	-	16 (15.68)	-
Length of ICU stay (days)	-	3.13 ± 7.18	-

BMI: Body Mass Index; LCS: Long-COVID syndrome; ICU: Intensive Care Unit. Results are expressed as mean ± SD. * *p* < 0.05, ** *p* < 0.001.

**Table 2:** Symptoms severity, functional disability, and global health perceived between groups.

Variables	LCS Non-Hospitalized Group (n = 363)			LCS Hospitalized Group (n = 102)			*p* between Groups
	Pre-COVID	Now	*p*-Value	Pre-COVID	Now	*p*-Value	
Breathlessness at rest	1.19 ± 2.95	3.27 ± 2.81	<0.001 **	1.48 ± 3.15	5.04 ± 2.65	<0.001 **	<0.001 **
Breathlessness dressing	1.14 ± 2.83	3.56 ± 2.92	<0.001 **	1.28 ± 2.92	5.39 ± 2.48	<0.001 **	<0.001 **
Breathlessness stairs	2.00 ± 2.81	6.09 ± 2.88	<0.001 **	2.05 ± 2.78	7.29 ± 2.77	<0.001 **	<0.001 **
Pain	1.08 ± 1.81	6.51 ± 2.68	<0.001 **	0.93 ± 1.33	7.29 ± 1.83	<0.001 **	0.001 *
Fatigue	1.90 ± 2.15	7.43 ± 2.51	<0.001 **	1.71 ± 1.98	8.09 ± 1.80	<0.001 **	0.003 *
Anxiety	1.39 ± 1.94	4.16 ± 3.22	<0.001 **	1.06 ± 1.45	4.96 ± 3.12	<0.001 **	0.026 *
Depression	0.82 ± 1.68	3.18 ± 3.34	<0.001 **	0.56 ± 1.38	4.05 ± 3.02	<0.001 **	0.013 *
Mobility	0.73 ± 1.87	4.59 ± 2.91	<0.001 **	0.68 ± 1.67	5.78 ± 2.36	<0.001 **	<0.001 **
Personal care	0.32 ± 1.17	2.85 ± 2.91	<0.001 **	0.28 ± 1.01	4.26 ± 2.83	<0.001 **	<0.001 **
Usual activities	0.76 ± 1.76	6.80 ± 2.72	<0.001 **	0.76 ± 1.90	7.67 ± 1.79	<0.001 **	<0.001 **
Global health perceived	7.45 ± 2.76	4.14 ± 2.48	<0.001 **	7.25 ± 2.77	3.18 ± 1.98	<0.001 **	<0.001 **

LCS: Long-COVID syndrome. Results are expressed as mean ±SD. * *p* < 0.05, ** *p* < 0.001.

**Table 3:** Additional symptoms between groups.

Variables	LCS Non-Hospitalized Group (n = 363)	LCS Hospitalized Group (n = 102)	*p*
Laryngeal complications n (%)	219 (60.33)	76 (74.51)	0.008 *
Voice complications n (%)	177 (48.76)	68 (66.67)	0.002 *
Swallowing complications n (%)	127 (34.99)	49 (48.04)	0.021 *
Nutritional alterations n (%)	176 (48.48)	47 (46.08)	0.737
Bowel incontinence n (%)	152 (41.87)	52 (50.98)	0.114
Bladder incontinence n (%)	112 (30.85)	31 (30.39)	1.000
Concentration difficulties n (%)	321 (88.43)	101 (99.02)	<0.001 **
Memory difficulties n (%)	312 (85.95)	98 (96.08)	0.005 *
Communication difficulties n (%)	269 (74.10)	91 (89.22)	0.001 *
Labor difficulties n (%)	317 (87.33)	101 (99.02)	<0.001 **

LCS: Long-COVID syndrome. Results are presented as n (%). * *p* < 0.05, ** *p* < 0.005.

## Data Availability

No additional data are available.

## References

[1] Fernández-de-Las-Peñas C., Palacios-Ceña D., Gómez-Mayordomo V., Cuadrado M.L., Florencio L.L. (2021). Defining Post-COVID Symptoms (Post-Acute COVID, Long COVID, Persistent Post-COVID): An Integrative Classification. Int. J. Environ. Res. Public Health.

[2] Haleem A., Javaid M., Vaishya R. (2020). Effects of COVID-19 pandemic in daily life. Curr. Med. Res. Pract..

[3] Remuzzi A., Remuzzi G. (2020). COVID-19 and Italy: What next?. Lancet.

[4] Alimohamadi Y., Sepandi M., Taghdir M., Hosamirudsari H. (2020). Determine the most common clinical symptoms in COVID-19 patients: A systematic review and meta-analysis. J. Prev. Med. Hyg..

[5] Chu D., Chen R.C., Ku C.Y., Chou P. (2008). The impact of SARS on hospital performance. BMC Health Serv. Res..

[6] Kira I.A., Alpay E.H., Ayna Y.E., Shuwiekh H.A.M., Ashby J.S., Turkeli A. (2022). The effects of COVID-19 continuous traumatic stressors on mental health and cognitive functioning: A case example from Turkey. Curr. Psychol..

[7] Hui D.S., Joynt G.M., Wong K.T., Gomersall C.D., Li T.S., Antonio G., Sung J.J.Y. (2005). Impact of severe acute respiratory syndrome (SARS) on pulmonary function, functional capacity and quality of life in a cohort of survivors. Thorax.

[8] van Walraven C., Forster A.J. (2007). Anticoagulation control in the peri-hospitalization period. J. Gen. Intern. Med..

[9] Davidson J.E., Jones C., Bienvenu O.J. (2012). Family response to critical illness: Postintensive care syndrome-family. Crit. Care Med..

[10] Bergquist S.H., Partin C., Roberts D.L., O’Keefe J.B., Tong E.J., Zreloff J., Moore M.A. (2020). Non-hospitalized Adults with COVID-19 Differ Noticeably from Hospitalized Adults in Their Demographic, Clinical, and Social Characteristics. SN Compr. Clin. Med..

[11] Holshue M.L., DeBolt C., Lindquist S., Lofy K.H., Wiesman J., Bruce H., Pillai S.K. (2020). First case of 2019 novel coronavirus in the United States. N. Engl. J. Med..

[12] Yip Y.C., Yip K.H., Tsui W.K. (2022). Psychological experiences of patients with coronavirus disease 2019 (COVID-19) during and after hospitalization: A descriptive phenomenological study. Int. J. Environ. Res. Public Health.

[13] Fernández-de-las-Peñas C. (2022). Long COVID: Current definition. Infection.

[14] Soriano J.B., Murthy S., Marshall J.C., Relan P., Diaz J.V. (2022). WHO Clinical Case Definition Working Group on Post-COVID-19 Condition. A clinical case definition of post-COVID-19 condition by a Delphi consensus. Lancet Infect. Dis..

[15] Rubin R. (2020). As Their Numbers Grow, COVID-19 “Long Haulers” Stump Experts. JAMA.

[16] Ojka A., Machniak M., Andrzejewski W., Kosendiak A., Chwałczyńska A. (2022). Changes in physical activity and the occurrence of specific symptoms of “Long-COVID syndrome” in men aged 18–25. Int. J. Environ. Res. Public Health.

[17] Nabavi N. (2020). Long covid: How to define it and how to manage it. BMJ.

[18] Garg P., Arora U., Kumar A., Wig N. (2021). The “post-COVID” syndrome: How deep is the damage?. J. Med. Virol..

[19] Lopez-Leon S., Wegman-Ostrosky T., Perelman C., Sepulveda R., Rebolledo P.A., Cuapio A., Villapol S. (2021). More than 50 Long-term effects of COVID-19: A systematic review and meta-analysis. Sci. Rep..

[20] Fernández-de-Las-Peñas C., Palacios-Ceña D., Gómez-Mayordomo V., Florencio L.L., Cuadrado M.L., Plaza-Manzano G., Navarro-Santana M. (2021). Prevalence of post-COVID-19 symptoms in hospitalized and non-hospitalized COVID-19 survivors: A systematic review and meta-analysis. Eur. J. Intern. Med..

[21] Schweizer C., Edwards R.D., Bayer-Oglesby L., Gauderman W.J., Ilacqua V., Juhani Jantunen M., Künzli N. (2007). Indoor time–microenvironment–activity patterns in seven regions of Europe. J. Expo. Sci. Environ. Epidemiol..

[22] Rothe C., Schunk M., Sothmann P., Bretzel G., Froeschl G., Wallrauch C., Hoelscher M. (2020). Transmission of 2019-nCoV Infection from an Asymptomatic Contact in Germany. N. Engl. J. Med..

[23] Dyal J.W. (2020). COVID-19 Among Workers in Meat and Poultry Processing Facilities—19 States, April 2020. MMWR Morb. Mortal Wkly. Rep..

[24] Park S.Y., Kim Y.-M., Yi S., Lee S., Na B.-J., Kim C.B., Jeong E.K. (2020). Coronavirus Disease Outbreak in Call Center, South Korea. Emerg. Infect. Dis..

[25] What Might Long COVID Mean for the Nation’s Health? The Health Foundation. https://www.health.org.uk/news-and-comment/blogs/what-might-long-covid-mean-for-the-nations-health.

[26] Office for National Statistics Prevalence of Ongoing Symptoms Following Coronavirus (COVID-19) Infection in the UK. https://www.ons.gov.uk/peoplepopulationandcommunity/healthandsocialcare/conditionsanddiseases/bulletins/prevalenceofongoingsymptomsfollowingcoronaviruscovid19infectionintheuk/1july2021.

[27] Giorgi G., Lecca L.I., Alessio F., Finstad G.L., Bondanini G., Lulli L.G., Mucci N. (2020). COVID-19-related mental health effects in the workplace: A narrative review. Int. J. Environ. Res. Public Health.

[28] Bakker A.B., Demerouti E. (2017). Job demands-resources theory: Taking stock and looking forward. J. Occup. Health Psychol..

[29] Bellotti L., Zaniboni S., Balducci C., Grote G. (2021). Rapid Review on COVID-19, Work-Related Aspects, and Age Differences. Int. J. Environ. Res. Public Health.

[30] Lai J., Ma S., Wang Y., Cai Z., Hu J., Wei N., Hu S. (2020). Factors associated with mental health outcomes among health care workers exposed to coronavirus disease 2019. JAMA Netw. Open.

[31] Chew N.W., Lee G.K., Tan B.Y., Jing M., Goh Y., Ngiam N.J., Sharma V.K. (2020). A multinational, multicentre study on the psychological outcomes and associated physical symptoms amongst healthcare workers during COVID-19 outbreak. Brain Behav. Immun..

[32] D’Ettorre G., Ceccarelli G., Santinelli L., Vassalini P., Innocenti G.P., Alessandri F., Tarsitani L. (2021). Post-Traumatic Stress Symptoms in Healthcare Workers Dealing with the COVID-19 Pandemic: A Systematic Review. Int. J. Environ. Res. Public Health..

[33] Salazar de Pablo G., Serrano J.V., Catalan A., Arango C., Moreno C., Ferre F., Fusar-Poli P. (2020). Impact of coronavirus syndromes on physical and mental health of health care workers: Systematic review and meta-analysis. J. Affect. Disord..

[34] Michelen M., Manoharan L., Elkheir N., Cheng V., Dagens A., Hastie C., Stavropoulou C. (2021). Characterising long COVID: A living systematic review. BMJ Glob. Health.

[35] Pandey B., Gautam S., Karki S., Lama M., Sigdel K.R., Hirachan N. (2022). Prevalence of Long covid-19 syndrome among health care workers of Patan Academy of Health Sciences. J. Patan Acad. Health Sci..

[36] Ayuso García B., Besteiro Balado Y., Pérez López A., Romay Lema E., Marchán-López Á., Rodríguez Álvarez A., Rabuñal Rey R. (2023). Assessment of post-COVID symptoms using the C19-YRS tool in a cohort of patients from the first pandemic wave in northwestern Spain. Telemed. J. E-Health.

[37] Sivan M., Parkin A., Makower S., Greenwood D.C. (2022). Post-COVID syndrome symptoms, functional disability, and clinical severity phenotypes in hospitalized and nonhospitalized individuals: A cross-sectional evaluation from a community COVID rehabilitation service. J. Med. Virol..

[38] von Elm E., Altman D.G., Egger M., Pocock S.J., Gøtzsche P.C., Vandenbroucke J.P. (2014). The strengthening the reporting of observational studies in epidemiology (STROBE) statement: Guidelines for reporting observational studies. Int. J. Surg..

[39] World Medical Association (2013). World Medical Association declaration of Helsinki: Ethical principles for medical research involving human subjects. JAMA-J. Am. Med. Assoc..

[40] Troyano-Hernáez P., Reinosa R., Holguín Á. (2022). Evolution of SARS-CoV-2 in Spain during the first two years of the pandemic: Circulating variants, amino acid conservation, and genetic variability in structural, non-structural, and accessory proteins. Int. J. Mol. Sci..

[41] Del Águila-Mejía J., Wallmann R., Calvo-Montes J., Rodríguez-Lozano J., Valle-Madrazo T., Aginagalde-Llorente A. (2022). Secondary attack rate, transmission and incubation periods, and serial interval of SARS-CoV-2 Omicron variant, Spain. Emerg. Infect. Dis..

[42] NHS England (2021). National Guidance for Post-COVID Syndrome Assessment Clinics.

[43] National Institute for Health and Care Excellence (2021). COVID-19 Rapid Guideline: Managing the Long-Term Effects of COVID-19. https://www.nice.org.uk/guidance/ng188/chapter/common-symptoms-of-ongoing-symptomatic-covid-19-and-post-covid-19-syndrome#common-symptoms-of-ongoing-symptomatic-covid-19-and-post-covid-19-syndrome.

[44] Sivan M., Preston N., Parkin A., Makower S., Gee J., Ross D., Horton M. (2022). The modified COVID-19 Yorkshire Rehabilitation Scale (C19-YRSm) patient-reported outcome measure for Long COVID or Post-COVID-19 syndrome. J. Med. Virol..

[45] Jacobs L.G., Gourna Paleoudis E., Lesky-Di Bari D., Nyirenda T., Friedman T., Gupta A., Aschner J.L. (2020). Persistence of symptoms and quality of life at 35 days after hospitalization for COVID-19 infection. PLoS ONE.

[46] Sun P., Qie S., Liu Z., Ren J., Li K., Xi J. (2020). Clinical characteristics of hospitalized patients with SARS-CoV-2 infection: A single arm meta-analysis. J. Med. Virol..

[47] Cholankeril G., Podboy A., Aivaliotis V.I., Pham E.A., Spencer S.P., Kim D., Ahmed A. (2020). Association of digestive symptoms and hospitalization in patients with SARS-CoV-2 infection. medRxiv.

[48] Halpin S., O’Connor R., Sivan M. (2021). Long COVID and chronic COVID syndromes. J. Med. Virol..

[49] Torres-Castro R., Vasconcello-Castillo L., Alsina-Restoy X., Solis-Navarro L., Burgos F., Puppo H., Vilaró J. (2021). Respiratory function in patients post-infection by COVID-19: A systematic review and meta-analysis. Pulmonology.

[50] Mulchandani R., Lyngdoh T., Kakkar A.K. (2021). Deciphering the COVID-19 cytokine storm: Systematic review and meta-analysis. Eur. J. Clin. Investig..

[51] Douaud G., Lee S., Alfaro-Almagro F., Arthofer C., Wang C., McCarthy P., Smith S.M. (2022). SARS-CoV-2 is associated with changes in brain structure in UK Biobank. Nature.

[52] Díez-Cirarda M., Yus M., Gómez-Ruiz N., Polidura C., Gil-Martínez L., Delgado-Alonso C., Matias-Guiu J.A. (2023). Multimodal neuroimaging in post-COVID syndrome and correlation with cognition. Brain.

[53] Khoja O., Silva Passadouro B., Mulvey M., Delis I., Astill S., Tan A.L., Sivan M. (2022). Clinical Characteristics and Mechanisms of Musculoskeletal Pain in Long COVID. J. Pain. Res..

[54] Soares F.H.C., Kubota G.T., Fernandes A.M., Hojo B., Couras C., Costa B.V., Lapa J.D.D.S., Braga L.M., Almeida M.M., Cunha P.H.M.D. (2021). Prevalence and characteristics of new-onset pain in COVID-19 survivours, a controlled study. Eur. J. Pain..

[55] Calvache-Mateo A., López-López L., Martín-Núñez J., Heredia-Ciuró A., Granados-Santiago M., Ortiz-Rubio A., Valenza M.C. (2023). Pain and Clinical Presentation: A Cross-Sectional Study of Patients with New-Onset Chronic Pain in Long-COVID-19 Syndrome. Int. J. Environ. Res. Public Health.

[56] Morin L., Savale L., Pham T., Colle R., Figueiredo S., Harrois A., Gasnier M., Lecoq A.L., Meyrignac O., Noel N. (2021). Four-Month Clinical Status of a Cohort of Patients After Hospitalization for COVID-19. JAMA.

[57] Blomberg B., Mohn K.G., Brokstad K.A., Zhou F., Linchausen D.W., Hansen B.A., Langeland N. (2021). Long COVID in a prospective cohort of home-isolated patients. Nat. Med..

[58] Keijsers K., Broeders M., Baptista Lopes V., Klinkert A., van Baar J., Nahar-van Venrooij L., Kerckhoffs A. (2022). Memory impairment and concentration problems in COVID-19 survivors 8 weeks after non-ICU hospitalization: A retrospective cohort study. J. Med. Virol..

[59] Merad M., Blish C.A., Sallusto F., Iwasaki A. (2022). The immunology and immunopathology of COVID-19. Science.

[60] Robinson-Agramonte M.A., Gonçalves C.A., Noris-García E., Préndes Rivero N., Brigida A.L., Schultz S., García R.J.G. (2021). Impact of SARS-CoV-2 on neuropsychiatric disorders. World J. Psychiatry.

[61] Seeßle J., Waterboer T., Hippchen T., Simon J., Kirchner M., Lim A., Merle U. (2022). Persistent Symptoms in Adult Patients 1 Year After Coronavirus Disease 2019 (COVID-19): A Prospective Cohort Study. Clin. Infect. Dis..

[62] Muñoz-Corona C., Gutiérrez-Canales L.G., Ortiz-Ledesma C., Martínez-Navarro L.J., Macías A.E., Scavo-Montes D.A., Guaní-Guerra E. (2022). Quality of life and persistence of COVID-19 symptoms 90 days after hospital discharge. J. Int. Med. Res..

[63] Korkut S., Ülker T. (2022). The Effect of Pain Experienced During the COVID-19 Infection on the Fear of Pain and Quality of Life. Pain. Manag. Nurs. Off. J. Am. Soc. Pain. Manag. Nurses.

[64] Pires R.E., Reis I.G.N., Waldolato G.S., Pires D.D., Bidolegui F., Giordano V. (2022). What Do We Need to Know About Musculoskeletal Manifestations of COVID-19?: A Systematic Review. JBJS Rev..

